# Akt/AS160 Signaling Pathway Inhibition Impairs Infection by Decreasing Rab14-Controlled Sphingolipids Delivery to Chlamydial Inclusions

**DOI:** 10.3389/fmicb.2019.00666

**Published:** 2019-04-03

**Authors:** Anahí Capmany, Julián Gambarte Tudela, Mariano Alonso Bivou, María T. Damiani

**Affiliations:** ^1^Laboratorio de Bioquímica e Inmunidad, Área de Química Biológica, Facultad de Ciencias Médicas, Universidad Nacional de Cuyo, Mendoza, Argentina; ^2^Consejo Nacional de Investigaciones Científicas y Técnicas, Mendoza, Argentina; ^3^Instituto de Medicina y Biología Experimental de Cuyo, Consejo Nacional de Investigaciones Científicas y Técnicas, Mendoza, Argentina

**Keywords:** *Chlamydia trachomatis*, Akt/AS160 signaling pathway, Rab14, Golgi-derived sphingolipids, Rab proteins, GTPase activating proteins, vesicular transport

## Abstract

*Chlamydia trachomatis*, an obligate intracellular bacterium, intercepts different trafficking pathways of the host cell to acquire essential lipids for its survival and replication, particularly from the Golgi apparatus via a Rab14-mediated transport. Molecular mechanisms underlying how these bacteria manipulate intracellular transport are a matter of intense study. Here, we show that *C. trachomatis* utilizes Akt/AS160 signaling pathway to promote sphingolipids delivery to the chlamydial inclusion through Rab14-controlled vesicular transport. *C. trachomatis* provokes Akt phosphorylation along its entire developmental life cycle and recruits phosphorylated Akt (pAkt) to the inclusion membrane. As a consequence, Akt Substrate of 160 kDa (AS160), also known as TBC1D4, a GTPase Activating Protein (GAP) for Rab14, is phosphorylated and therefore inactivated. Phosphorylated AS160 (pAS160) loses its ability to promote GTP hydrolysis, favoring Rab14 binding to GTP. Akt inhibition by an allosteric isoform-specific Akt inhibitor (iAkt) prevents AS160 phosphorylation and reduces Rab14 recruitment to chlamydial inclusions. iAkt further impairs sphingolipids acquisition by *C. trachomatis-*inclusion and provokes lipid retention at the Golgi apparatus. Consequently, treatment with iAkt decreases chlamydial inclusion size, bacterial multiplication, and infectivity in a dose-dependent manner. Similar results were found in AS160-depleted cells. By electron microscopy, we observed that iAkt generates abnormal bacterial forms as those reported after sphingolipids deprivation or Rab14 silencing. Taken together, our findings indicate that targeting the Akt/AS160/Rab14 axis could constitute a novel strategy to limit chlamydial infections, mainly for those caused by antibiotic-resistant bacteria.

## Introduction

*Chlamydia trachomatis* (*C. trachomatis*), a Gram-negative obligate intracellular pathogen, is the most frequent cause of bacterial sexually transmitted diseases worldwide (WHO, 2014). Chlamydial infections are a serious problem in reproductive health. Most of the cases are asymptomatic, which lead to undiagnosed and untreated infections. Consequently, a chronic course is the common feature underlying inflammation and scarring responsible for undesirable complications such as tubal obstruction, ectopic pregnancy and infertility among young women (Luján et al., 2016; Tsevat et al., 2017).

*Chlamydia trachomatis* invades human cervical epithelial cells through different receptors and displays effective molecular mechanisms to promote its internalization (Clifton et al., 2004; Cossé et al., 2016; Lujan et al., 2018). Once inside the host cell, *C. trachomatis* replicates in a modified vacuole called inclusion. Its biphasic developmental cycle involves two bacterial forms: infectious non-replicative elementary bodies (EBs), and non-infectious replicant-competent metabolically active reticulate bodies (RBs) (Bastidas et al., 2013; Vromman et al., 2014). Under stressing conditions, such as sustained treatment with penicillin (Cevenini et al., 1988), gamma-interferon (INFγ) (Beatty et al., 1993, 1994a) and nutrients or sphingolipids deprivation (Raulston, 1997; Robertson et al., 2009; Capmany and Damiani, 2010), reticulate bodies acquire abnormal characteristics. Aberrant bacteria (AB) are non-infectious non-replicative forms with low metabolic activity. AB can remain inside host cells for years, being typical of persistent chlamydial infections associated with detrimental consequences for human fertility (Beatty et al., 1994b; Witkin et al., 2017). When stressing conditions disappear, AB re-enter the normal developmental cycle. After several rounds of asynchronous replication, RBs differentiate back into EBs. Finally, bacteria are released either by host cell lysis or extrusion of inclusions to spread the infection to neighboring cells (Hybiske and Stephens, 2007).

*Chlamydia trachomatis* has developed multiple strategies to manipulate molecular controllers of intracellular trafficking to escape from the degradative phagocytic pathway and to piracy biosynthetic routes (Hackstadt et al., 1995; Damiani et al., 2014). This way, *C. trachomatis* creates a favorable environment that helps it to avoid immune responses (Cunha and Zamboni, 2013; Rajeeve et al., 2018) and, simultaneously allows the acquisition of needed host molecules for bacterial growth and replication (Saka and Valdivia, 2010). *C. trachomatis* obtains amino acids, nucleotides, and lipids from the host cell (van Ooij et al., 2000; Saka and Valdivia, 2010; Mehlitz et al., 2017). Particularly, sphingomyelin, cholesterol, and neutral lipids are obtained by hijacking TGN-derived vesicles (Hackstadt et al., 1995, 1996; Carabeo et al., 2003), multivesicular bodies (Beatty, 2006, 2008; Gambarte Tudela et al., 2015) and lipid droplets, respectively (Kumar et al., 2006; Cocchiaro et al., 2008).

Rab proteins, a family of small GTPases, play a key role in the regulation of vesicular transport (Pfeffer, 2017); and some of these enzymes have been implicated in chlamydial inclusion development (Damiani et al., 2014). *C. trachomatis* recruits Rab1, Rab4, Rab6, Rab11, Rab14, and Rab39a to the inclusion membrane (Rzomp et al., 2003, 2006; Rejman Lipinski et al., 2009; Capmany and Damiani, 2010; Leiva et al., 2013; Gambarte Tudela et al., 2015). We have demonstrated that Rab14, which controls transport from the Golgi apparatus to early endosomes and the plasma membrane, is involved in sphingolipids delivery to the chlamydial inclusion (Capmany and Damiani, 2010; Capmany et al., 2011).

Rab GTPases are active in the GTP-bound state and inactive when associating to GDP (Pfeffer, 2017). Several proteins assist Rabs in their GTP/GDP cycling: (i) Guanine nucleotide Exchange Factors (GEFs) that exchange GDP for GTP, turning Rabs into their active state; and (ii) GTPase Activating Proteins (GAPs) which increase the intrinsic hydrolytic activity of Rab proteins, favoring the inactive GDP-bound state (Zerial and McBride, 2001). Phosphatidylinositol-3-kinase (PI3K)/Akt/AS160 signaling pathway arises as a novel regulator of Rab GTP/GDP cycling. It has been extensively studied in adipocytes and muscle cells (Klip et al., 2014). Akt kinase, upon activation, phosphorylates and inactivates AS160 which is GAP for Rab2, Rab8A, Rab10, and Rab14 (Mîinea et al., 2005; Ishikura et al., 2006). Consequently, Rab proteins hydrolyze GTP more slowly and remain in the active state for longer time. The link between Akt/AS160 pathway and Rab function has been well described in the transport of GLUT4-containing vesicles to the plasma membrane after insulin stimulus (Klip et al., 2014) and in aquaporin-2 translocation to the membrane of renal collecting ducts (Jung and Kwon, 2010). Noteworthy, some bacteria like *Salmonella typhi* usurps PI3K/Akt pathway to avoid fusion with lysosomes by altering Rab proteins function, and at the same time, to control actin dynamics mediated by RhoA and Rac1 during bacteria internalization (Kuijl et al., 2007).

*Chlamydia trachomatis* resides inside the boundaries of the inclusion, from where it actively interacts with the host cell by exporting various effector proteins via a type three secretion system, either into host cytoplasm or integrated into the inclusion membrane. Some of these bacterial proteins -like translocated early phospho-protein (TepP) and other still unidentified factors- target PI3K signaling pathway to control glucose metabolism, cell growth and proliferation, genome stability and transcription, and survival of host cell (Verbeke et al., 2006; Rajalingam et al., 2008; Siegl et al., 2014; Subbarayal et al., 2015; Carpenter et al., 2017). Downstream of PI3K is the serine/threonine kinase Akt, also known as protein kinase B, whose activation is controlled by two main phosphorylation sites: Thr-308 and Ser-473 (Alessi et al., 1997; Sarbassov, 2005). Several reports described PI3K/Akt involvement in different aspects of chlamydial infection like invasion (Lane et al., 2008; Patel et al., 2014; Subbarayal et al., 2015), apoptosis resistance and bacterial development (Verbeke et al., 2006; Rajalingam et al., 2008; Gurumurthy et al., 2010; Subbarayal et al., 2015). Akt participates in multiple signaling pathways (Huang et al., 2018), however, despite advances achieved to unravel its role in chlamydial infection, many gaps remain unsolved. Here, we demonstrate a link between Akt activation and intracellular transport in *C. trachomatis*-infected cells. The bacterium hijacks Akt/AS160 pathway as a strategy to ensure the recruitment of Rab14-positive vesicles to chlamydial inclusions for seizing Golgi-derived sphingolipids. Accordingly, Akt inhibition restores AS160 GAP activity, which restricts Rab14-mediated sphingolipids delivery to chlamydial inclusions, limiting inclusion growth, bacterial replication and the yield of infectious progeny.

## Materials and Methods

### Cells and Bacteria

HeLa 229 cells (ABAC, Bs.As., Argentina) were grown in high glucose Dulbecco’s modified Eagle’s medium (D-MEM) (GIBCO-BRL, Bs.As., Argentina) supplemented with 10% fetal bovine serum (FBS) (Internegocios SA, Bs. As., Argentina). Cells were routinely checked for *Mycoplasma* contamination by PCR (van Kuppeveld et al., 1992). *C. trachomatis* serovar L2 434/Bu (*Ct*) (gently given and typified by Unidad de Estudios de *Chlamydias*, FFyB, UBA, Bs. As., Argentina) and *C. trachomatis* strain harboring p2TK2-SW2 IncDProm-RSGFP-IncDTerm serovar L2 (GFP-*Ct*) (kindly provided by Isabelle Derré) (Agaisse and Derré, 2013) were used. For bacterial propagation, HeLa cells were infected at a multiplicity of infection (MOI) of 20 and incubated at 37°C in an atmosphere of 5% CO_2_ and 95% humidified air for 48 h. Then, infected cells were scrapped in Hanks medium (0.137 M NaCl, 5.4 mM KCl, 0.25 mM Na_2_HPO_4_, 0.44 mM KH_2_PO_4_, 1.3 mM CaCl_2_, 1.0 mM MgSO_4_, 4.2 mM NaHCO_3_). The cell suspension obtained was incubated in an ultrasound bath for 5 min. Subsequently, the lysate was centrifuged at 4°C for 15 min at 500 rpm to remove cell debris. EBs in the supernatant were purified as previously described (Capmany and Damiani, 2010). Purified EBs were suspended in 0.2 M sucrose-5% FBS-0.02 M phosphate buffer (pH = 7.2) and titered by assessing the number of inclusion forming units (IFUs) per microliter as previously described (Capmany and Damiani, 2010).

### Antibodies, Plasmids, and Reagents

In this study, we used the following antibodies: rabbit polyclonal anti-MOMP coupled to FITC (DakoCytomation, Ely, United Kingdom); mouse monoclonal anti-MOMP (Santa Cruz); rabbit polyclonal anti-CT529 (gently provided by Agathe Subtil, Pasteur Institute, Paris, France); rabbit polyclonal anti-Rab14 (Abcam, ab40938, United States); mouse monoclonal anti-Rab14 (Santa Cruz Biotechnology, sc-271401, United States); rabbit polyclonal anti-Akt (pan) (Cell Signaling, 4685, United States); rabbit polyclonal anti-phosphorylated Akt (Ser-473) (Cell Signaling, 4060, United States); rabbit polyclonal anti-AS160 (pan) (Cell Signaling, 26705, United States); rabbit polyclonal anti-phosphorylated AS160 (Ser-318) (Cell Signaling, 8619, United States); mouse monoclonal anti-GM130 (BD Biosciences 610823, United States); mouse monoclonal anti-actin (Abcam, ab3280, United States); mouse monoclonal anti-clathrin (Santa Cruz, United States); goat anti-mouse HRP-conjugated IgG and goat anti-rabbit HRP-conjugated IgG, donkey anti-rabbit Cy5-labeled IgG, donkey anti-mouse Cy3- or Cy5-labeled IgG, goat anti-rabbit Cy3-, Cy5- or FITC-labeled IgG (Jackson ImmunoResearch Laboratories, West Grove, PA, United States and Invitrogen, Bs. As. Argentina). Plasmids used were pEGFP, pEGFP-Rab14 WT (gently provided by Dr. Alfred Nordheim, University of Tuebingen, Tuebingen, Germany), pCherry-Rab14 WT (subcloned at our lab) and pEGFP-RUFY1 (kindly given by Dr. Shin, Faculty of Pharmaceutical Sciences, Kyoto University, Japan). To inhibit Akt, infected cells were incubated with 2, 5, or 10 μM of Akt Inhibitor VIII Isozyme-Selective Akti-1/2 (iAkt) (Sigma, 124018, Bs. As., Argentina) for the indicated post-infection times (pi). Dimethyl sulfoxide (DMSO) (Merck, Bs. As., Argentina) was used for control conditions. Degradative compartments were visualized by incubation with DQ^TM^ bovine serum albumin (DQ-BSA) (Invitrogen, D12051, Bs. As., Argentina). Hoechst 33258, propidium iodide (PI) and DAPI were used for nucleic acids staining (Life Technologies, Bs. As., Argentina).

### Cell Transfection, Gene Silencing and Infection

HeLa 229 cells were grown on 12-mm-diameter glass coverslips in 24-well plates (ETC Internacional, Bs. As., Argentina) until 70% confluence. Cells were washed once with PBS and serum-free D-MEM (GIBCO-BRL Bs. As., Argentina) was added. Then, cells were transfected with Lipofectamine 2000 (Invitrogen, Bs. As., Argentina) using 1 μl per 1 μg of DNA per well according to the manufacturer’s protocol. At 24 h post-transfection, cells were infected with *C. trachomatis* (*Ct* or GFP-*Ct*; MOI 0.5–5). Bacteria were added to HeLa cells, plates were centrifuged for 15 min at 4°C at 1,000 rpm and then maintained for 2 h at 37°C. After that, cells were washed three times with PBS to eliminate non-internalized bacteria, and finally, cells were incubated in the presence of infection medium (D-MEM without antibiotics) at 37°C in an atmosphere of 5% CO_2_ and 95% humidified air for the indicated times (post-infection period).

To knock-down AS160, HeLa cells were transfected in either 6-well plates or 12-well plates with siRNA-AS160 or siRNA-Luciferase (control cells) (25 nM final concentration) (Thermo Scientific Dharmacon, J021230-10, United States) using Lipofectamine RNAi MAX Transfection Reagent (5 μL; Invitrogen, Bs. As., Argentina). At 24 or 48 h post-transfection, depending on the assay, cells were infected with *C. trachomatis*, as described above, and incubated for the indicated periods of time for further manipulations.

### Immunoblotting

For western blots, protein extracts were generated by lysing cells in the presence of protease inhibitor cocktail p-2714 (Sigma-Aldrich, United States). Protein levels were quantified by Bradford’s method (Tetrahedron, Bs. As., Argentina). Equal amounts of proteins were resolved in 12% acrylamide SDS-PAGE gels. Separated proteins were transferred onto 0.45 μm nitrocellulose membranes (Amersham, Germany) and incubated overnight with the corresponding primary antibodies followed by goat anti-rabbit HRP-conjugated IgG (1:5000). Primary antibodies were rabbit polyclonal anti-Akt (pan) (1:1000), rabbit polyclonal anti-phosphorylated Akt (Ser-473) (1:1000), rabbit polyclonal anti-AS160 (pan) (1:1000), rabbit polyclonal anti-phosphorylated AS160 (Ser-318) (1:1000). Protein loading was assessed with monoclonal mouse anti-actin (1:1000) or monoclonal mouse anti-clathrin (1:1000) followed by goat anti-mouse HRP-conjugated IgG (1:5000). An Amersham ECL Plus^TM^ kit was used to detect HRP activity (GE Healthcare Life Sciences, Bs. As., Argentina) in an ImageQuant LAS4000.

### Subcellular Fractionation

Cell fractionation assay protocol was modified from previous reports (Chua and Wong, 2013). Cells were harvested by trypsinization and washed with PBS. Then, cells were resuspended in 400 μl homogenizing buffer (8.6% saccharose, 25 mM HEPES pH 7.4, 5 mm MgCl, 1 mM EDTA, protease inhibitor 1x). Cells were lysed using 10 passes through a 22-gauge needle followed by 15 min incubation on ice. The homogenate was clarified by a 400 × *g* centrifugation for 5 min. The clarified homogenate was centrifuged at 100,000 × *g* for 2 h at 4°C (Optima TLX Ultracentrifuge, Beckmann Coulter). The cytosol fraction (supernatant) was separated from the membrane fraction (pellet) before the latter was resuspended with lysis buffer supplemented with protease inhibitors 1x. Both fractions were sonicated three times in a 5-s pulse at 10 Amp on ice. Cytosol and membrane fractions were incubated on ice for 1 h before a final centrifugation of 16,000 × *g* for 15 min. Clarified supernatants were subjected to protein quantification before WB analysis.

### Immunofluorescence and Confocal Microscopy

For immunofluorescence staining, formaldehyde-fixed cells were washed three times with PBS, incubated with 50 mM NH_4_Cl and permeabilized in PBS 0.2%/BSA 0.05%/saponin. Cells were then incubated with the primary antibody for 1 h, washed in PBS and incubated with FITC-, Cy3-or Cy5-coupled secondary antibodies (Jackson ImmunoResearch and Invitrogen). As for mounting medium 1 μg/mL Hoechst or DAPI/Mowiol (Molecular Probes, United States) was used. Immunofluorescence images were acquired by confocal microscopy using an Olympus FV FV-1000 and 10-ASW 1.7 software (Olympus, United States). Images were processed using Adobe Photoshop CS5, Adobe Illustrator CS5 (Adobe Systems, Inc., San Jose, CA, United States) and MacBiophotonics ImageJ.

### Sphingolipids Labeling and Quantification

Infected cells grown on 12-mm-diameter glass coverslips in 24-well plates were treated with iAkt for the indicated periods of time; and before its fixation, cells were incubated with 5 μM BODIPY TR ceramide-BSA complex in DMEM or BODIPY FL ceramide-BSA (Molecular Probes, United States) for 30 min at 4°C, then washed 3 times with PBS, and finally, maintained in DMEM without FBS for 30 min at 37°C. Confocal images were captured using the same parameters setting: equal optical magnification (606) and electronic zoom (26), identical laser potency (5%), identical photodetector gain (HV 480 V), identical scanning speed (12 ms/pixel). An Olympus FV-1000 spectral confocal unit mounted on an IX-25 Olympus inverted microscope was used. Confocal images were acquired at 512 by 512 pixels and analyzed with the FV10-ASW 1.7 Software (Olympus America, Inc., Melville, NY, United States). Fluorescence intensities of TR labeled lipids (635 nm laser) were determined by defining regions of interest (ROI) coincident with chlamydial inclusions (405 nm laser) or Golgi apparatus (delimited by GM130 labeling) by MacBiophotonic Image J and were expressed as arbitrary units (a.u.). Images were processed using Adobe Photoshop CS5 and Adobe Illustrator CS5 (Adobe Systems, Inc., San Jose, CA, United States).

### Thin Layer Chromatography of Lipids Extracts

HeLa cells were infected with *C. trachomatis* for 48 h. For the last 24 h of infection, cells were incubated in DMSO, or 2 μM or 10 μM iAkt. Cell lipid extraction and thin layer chromatography (TLC) were carried out as previously described (Capmany and Damiani, 2010). Before harvesting, cells were incubated for 30 min with BODIPY TR ceramide at 4°C in serum-free DMEM. Then, cells were washed with PBS and incubated with DMEM supplemented with FBS for 30 min at 37°C. Cells were subjected to Bligh and Dyer chloroform:methanol lipid extraction. A portion of the lipid samples were resuspended in 1:1 chloroform:methanol and fluorescently labeled lipids were quantified (530 nm excitation/620 nm emission, Packard FluoroCount^TM^ Microplate Fluorometer, United States). The remaining of the samples were resuspended in chloroform/methanol/HCl (100:100:1, v/v) and resolved on TLC plates using 1- butanol/methanol/acetic acid/water (8:2:1:2, v/v) as solvent system. Equal amounts of lipids were seeded. Fluorescent spots were visualized on air-dried plates upon 550 nm excitation in a LAS- 4000 EPUV luminometer and LAS image reader software (FUJI Life Science, Japan).

### Transmission Electron Microscopy

HeLa cells grown in T-25 mL flasks were infected with *C. trachomatis* L2 (*Ct*; MOI of 5) and treated with 5 μM iAkt from 2 h pi until its fixation at 24 h pi. Infected cell monolayers were fixed with 2% glutaraldehyde/PBS for 1 h at 37°C. Then, cells were removed with 1% gelatin/PBS, gently centrifuged (15 min at 1200 rpm) and washed three times with PBS. After that, cells were incubated with Osmium tetroxide/Potassium ferricyanide/PBS (1:1:1) for 90 min. Samples were dehydrated using increasing acetone series and embedded in Spurr’s resin (Ted Pella Inc., United States). Thin sections were cut with an ultramicrotome (Leica ultracut R, Austria) and stained with 1% uranyl acetate and Reynold’s lead citrate (Ted Pella Inc., United States) before they were observed with a Zeiss 900 electron microscope (Zeiss, Germany). Images were processed using Adobe Photoshop CS5 (Adobe Systems, Inc., San Jose, CA, United States).

### Flow Cytometry

Cell viability was assessed after iAkt treatment (10 μM, 24 h) or FBS starvation (4 h or 24 h) by incubation with zombie NIR fixable probe (BioLegend Co.) for 30 min as indicated in the manufacturer’s protocol. To evaluate bacterial replication, HeLa cells were infected with GFP-*Ct* and increasing concentrations of iAkt were added from 2 h pi until its fixation at 24 h pi. Cell-associated fluorescence was quantified in a BD FACSAria III (Becton Dickinson Biosciences). FlowJo^TM^ software was used to analyze data.

### Inclusion Forming Units

Inclusion Forming Units (IFUs) per microliter were assessed as previously described (Capmany and Damiani, 2010). Briefly, to analyze the effect of Akt inhibition on the yield of chlamydial infectious progeny, HeLa cells were infected with *C. trachomatis* and 2 h later, cells were treated with iAkt (2, 5, or 10 μM) or DMSO for the control condition. Cells were incubated for 48 h, lysed and scrapped in SPG buffer. Then, serial dilutions of EBs were inoculated onto HeLa cells seeded on 96-well plates. After 24 h, cells were fixed, permeabilized and stained with FITC-coupled anti-MOMP antibodies. Inclusions were visualized and counted in 30 fields using a T-2000 Nikon epifluorescence microscope (Nikon, Japan), and expressed as IFUs per μL.

### Statistical Analysis

Data were statistically analyzed by Student’s *t*-test, or one-way ANOVA and Bonferroni post-test, as indicated in each experiment (^∗^*p* < 0.05, ^∗∗^*p* < 0.01, ^∗∗∗^*p* < 0.001; ns, not significant).

## Results

### *C. trachomatis*-Induced Akt Phosphorylation Causes Its Intracellular Redistribution

**FIGURE 1:**
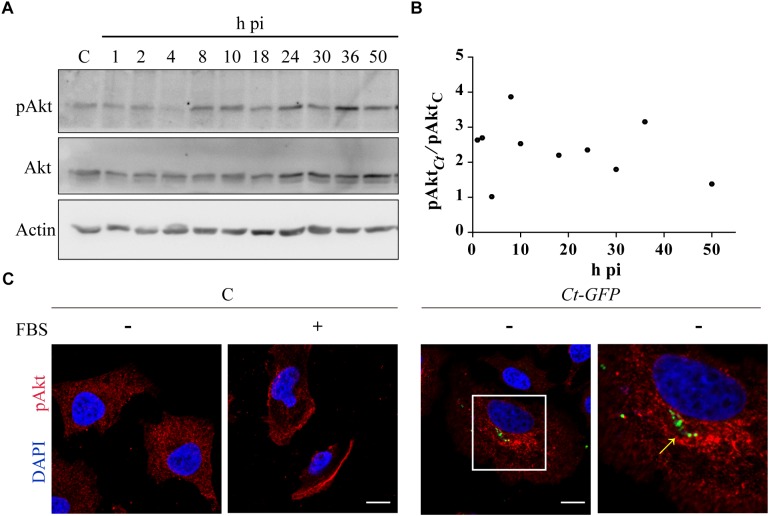
*Chlamydia trachomatis* (*Ct*) phosphorylates Akt and modifies its subcellular distribution. **(A)** HeLa cells were infected with *Ct* (MOI 1) for the indicated periods of time. Uninfected cells were used as control (C). Akt expression and phosphorylation was evaluated by immunoblot with anti-Akt and anti-pAkt antibodies, respectively. Actin was used as loading control. Cells were serum starved 4 h before sampling. Blots are representative of at least three independent experiments. **(B)** Relative phosphorylation level of Akt in *Ct*-infected cells compared to pAkt in uninfected cells. **(C)** HeLa cells were infected with *C. trachomatis* overexpressing the green fluorescence protein (*Ct*-GFP) (MOI 0.5) for 8 h and serum starved the last 4 h before fixation. Uninfected cells (C) incubated either with or without FBS served as control. Localization of phosphorylated Akt (pAkt) was detected by confocal microscopy with rabbit anti-pAkt (S473) followed by Cy3-coupled anti-rabbit antibodies. DNA was stained with DAPI. Arrow indicates chlamydial inclusions. Images are representative of three independent experiments.

Previous reports show discrepancy in Akt phosphorylation during infection, likely due to differences in *Chlamydia* species or serovars, experimental conditions or periods of infection (Verbeke et al., 2006; Lane et al., 2008; Rajalingam et al., 2008; Gurumurthy et al., 2010; Patel et al., 2014; Siegl et al., 2014; Subbarayal et al., 2015; Carpenter et al., 2017). Therefore, we monitored Akt expression and phosphorylation in *C. trachomatis*-infected cells along the whole bacterial developmental cycle. Akt and phosphorylated Akt (pAkt) were measured by western blot in a time course assay. To avoid Akt phosphorylation induced by components of FBS, most experiments have been performed after 4 h of FBS deprivation unless when used as positive control of Akt pathway activation (Watton and Downward, 1999). Cell viability was not affected by 4 h or 24 h of FBS starvation (Supplementary Figure S1). Although Akt phosphorylation and consequent activation occurred at every time post-infection (pi), we identified three main peaks of phosphorylation: at the beginning (2 h pi), at the middle (8 h pi), and at later stages (36 h pi) of the bacterial developmental cycle (Figure 1A). Figure 1B shows the ratio of phosphorylated Akt (pAkt) in *Chlamydia*-infected cells related to uninfected cells at different post-infection times. At the peaks, pAkt doubled or tripled the basal level of Akt phosphorylation in uninfected cells (Figure 1B). In contrast, total Akt expression levels remained unaltered after infection, regardeless of post-infection times (data not shown and Patel et al., 2014). Next, we assessed whether Akt intracellular distribution was altered. HeLa cells were infected with *C. trachomatis* and fixed at 8 h pi, at mid-stage Akt phosphorylation peak. Then, pAkt was detected by immunofluorescence using antibodies that recognize Akt phosphorylated on residue Ser-473. Remarkably, infection with *C. trachomatis* caused a dramatic redistribution of endogenous pAkt that concentrated at the perinuclear region where these bacteria localized, suggesting pAkt recruitment to chlamydial inclusion membranes (Figure 1C). Overall, Akt phosphorylation and association with inclusions supports the notion that it may play a role in chlamydial infection.

### An Allosteric Isoform-Specific Akt Inhibitor Reduces Chlamydial Inclusion Growth

**FIGURE 2:**
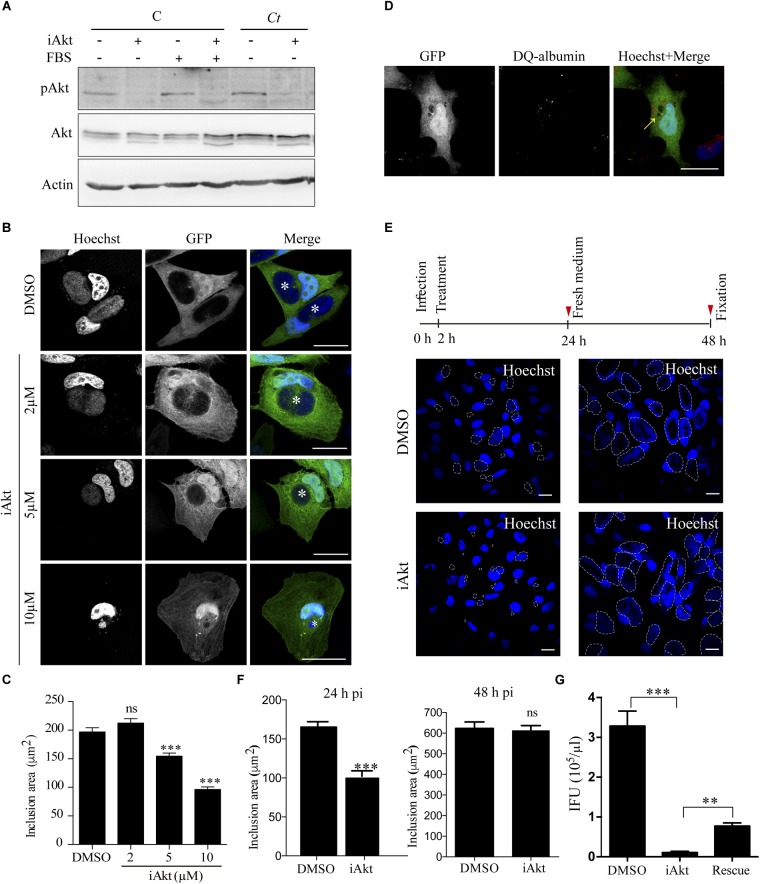
Inhibition of Akt impairs chlamydial inclusion growth. **(A)** Uninfected cells (C) incubated either in the presence or absence of serum (FBS) and serum starved *Ct*-infected cells (MOI 1) were treated with iAkt (10 μM) or DMSO the last 4 h before sampling at 8 h pi. Akt phosphorylation was evaluated by immunoblot. Actin was used as loading control. **(B)** GFP overexpresing HeLa cells were infected with *Ct* (MOI 1). At 2 h later, increasing concentrations of iAkt (2, 5, and 10 μM) or DMSO were added until fixation at 24 h pi. Inclusions are pointed out with asterisks. Bars represent 10 μm. **(C)** Quantification of chlamydial inclusion area of 100 cells from three independent experiments was performed with ImageJ. Data are the mean ± SEM (^∗∗∗^*p* < 0.001). **(D)** GFP overexpressing cells infected with *Ct* (MOI 2) were incubated with iAkt (10 μM) from 2 h pi until fixation at 24 h pi. DQ-albumin was added during the last 18 h of incubation. Images were analyzed by confocal microscopy. Arrow indicates chlamydial inclusions. Bars represent 10 μm. **(E)** Four sets of cells were infected with *Ct* (MOI 1). At 2 h pi, iAkt (5 μM) or DMSO were added to two plates each. At 24 h pi, a set of cells incubated with iAkt and a set incubated with DMSO were fixated and the remaining two sets of cells were washed out and incubated with fresh medium without drugs for an additional 24 h. DNA was stained with Hoechst. Inclusions are delimited with white dashed lines **(B,D,E)**. Bars represent 10 μm. **(F)** Quantification of the experiment above. Data are the mean ± SEM of three independent experiments (^∗∗∗^*p* < 0.001). **(G)** For a rescue assay, HeLa cells infected with *Ct* (MOI 1) were incubated with either DMSO (control cells) or 10 μM iAkt (iAkt and rescue). After 24 h pi, medium was renewed, and only in one set of cells was maintained with 10 μM iAkt (iAkt). At 48 h pi, EBs from all conditions (DMSO, iAkt and rescue) were harvested and infectious progeny was titrated in serial dilutions on HeLa cells. Bacteria were immunodetected with an anti-MOMP antibody coupled to FITC and inclusion forming units (IFU) were determined. Data are representative of two independent experiments performed in triplicates. Bars and the error bars represent mean ± SEM (^∗∗^*p* < 0.01; ^∗∗∗^*p* < 0.001).

To unravel the effect of Akt phosphorylation in the course of infection, we assayed an allosteric Akt inhibitor (iAkt) which is isoform-selective against Akt 1 and Akt 2 and possesses high specificity compared to ATP-competitive inhibitors that also target closely related kinases due to similarities in catalytic domains (Pentassuglia et al., 2016; Ebner et al., 2017; Mousset et al., 2018). The ability of iAkt to block the kinase phosphorylation and consequently its activation was assessed by western blot. Uninfected cells incubated with or without FBS, and *C. trachomatis*-infected cells maintained without FBS were treated with iAkt for 4h before pAkt detection (Figure 2A). It has been shown that the activation of Akt mediates actin remodeling required for the invasion process (Ganesan et al., 2004), therefore, we proved the efficiency of iAkt by infecting HeLa cells in presence or absence of 5 μM iAkt. Cells were fixed at 24 h pi and analyzed by confocal microscopy. Akt inhibition resulted in a significant decrease in bacterial internalization (Supplementary Figure S2). With the aim of analyzing the iAkt effect on *C. trachomatis* growth and development rather than in the number of invading bacteria, we decided to add the iAkt at 2 h pi in all the following experimental procedures in order to avoid interfering with the uptake process.

Briefly, green fluorescent protein (GFP)-overexpressing HeLa cells infected with *C. trachomatis* were incubated with increasing concentrations of iAkt until fixation at 24 h pi. Hoechst stained nuclei and bacterial DNA. Confocal images showed that as the concentration of Akt inhibitor increased, inclusions became smaller. GFP expression facilitated the visualization of chlamydial inclusions as black holes within infected cells since, inclusion membrane acts as a restrictive barrier for this soluble fluorescent protein (Boncompain et al., 2014). Maximal growth inhibition was achieved at 10 μM, reaching a decrease of approximately 50% in inclusion size (Figure 2B,C). These results further confirm the importance of Akt phosphorylation for bacterial development. By flow cytometry, we determined that host cell viability was unaltered after iAkt treatment, even at the higher doses of the inhibitor (10 μM, 24 h, 37°C) (Supplementary Figure S1). Alternatively, downsizing of inclusions by iAkt treatment may be attributed to direct drug toxicity to the bacteria causing their killing by targeting to the phagocytic pathway. To explore whether bacteria reached degradative compartments, we used a derivative of albumin called DQ-Albumin which acquires red fluorescence when it is hydrolyzed. HeLa cells overexpressing GFP were infected with *C. trachomatis* and treated with 10 μM of iAkt from 2 h pi until fixation at 24 h pi, DQ-albumin was added for the last 18 h of incubation. Confocal images show that in every cell, fluorescent albumin was not present within inclusions, indicating that even when bacterial growth was impaired, *C. trachomatis* was actively avoiding lysosomal or other types of degradation, since they remained inside a non-degradative vacuole (the inclusion). In addition to this, we found in every infected cell small vesicles likely lysosomes or late endosomes that preserved their degradative ability evinced by DQ-albumin red fluorescence, which served as a proper internal positive control (Figure 2D). Likely, iAkt was acting as a bacteriostatic agent rather than a bactericidal one at the low concentrations used. To analyze the reversibility of the iAkt effect on inclusion development, we incubated infected cells with 5 μM iAkt for 22 h and then removed the inhibitor and incubated the cells for additional 24 h in medium without the drug, allowing the inclusion to grow in case the bacteria within it were still alive. As it is shown in the images, there was a complete recovery of the size of the inclusions when the inhibitor was removed (Figure 2E,F). Chlamydial inclusion expansion precedes bacterial replication (Engström et al., 2015), therefore to directly assess bacterial viability and development after the strongest iAkt treatment (10 μM, 24 h, 37°C), we performed progeny/reinfection assays. Results show a significant recovery in the amount of infectious organisms after the removal of the inhibitor, notwithstanding, the inclusion forming units did not reach the levels of untreated cells (Figure 2G). On one hand, we determined that iAkt, at the concentrations assayed, does not cause bacterial death and that its effects are reversible. On the other hand, iAkt, acting as a bacteriostatic agent, reduces the chlamydial inclusion growth and bacterial replication in a dose-dependent manner.

### AS160 Is Inactivated by Akt in *Chlamydia*-Infected Cells

**FIGURE 3:**
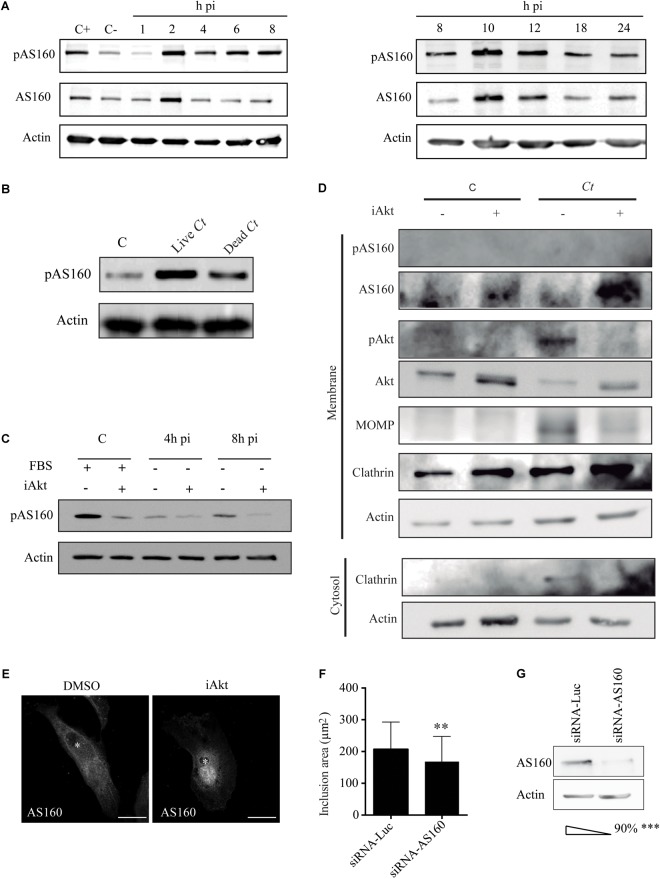
AS160 is phosphorylated by Akt during infection. **(A)** HeLa cells were infected with *Ct* (MOI 1) and serum-starved the last 4 h before sampling. AS160 expression and phosphorylation were evaluated by immunoblot with anti-AS160 and anti-pAS160 antibodies, respectively. Uninfected cells incubated in the absence (C–) or presence (C+) of FBS were used as a control. Actin indicates the protein load. Blots are representative of three independent experiments. **(B)** Serum-starved HeLa cells were incubated during 2 h with living or heat inactivated (10 min in boiling water) bacteria (MOI 5). Uninfected serum-starved cells were used as a control. AS160 phosphorylation was evaluated by immunoblot. Actin was used as loading control.**(C)** HeLa cells infected with *Ct* (MOI 1) for 4 h or 8 h, incubated in DMEM without FBS were treated with DMSO or iAkt (10 μM) the last 4 h of the experimental period. As control, FBS-stimulated uninfected HeLa cells were treated with DMSO or iAkt for 4 h. AS160 phosphorylation was evaluated by immunoblot. Actin was used as loading control. Blots are representative of three independent experiments. **(D)** Infected (24 h pi, MOI 3) and uninfected cells were treated with DMSO or iAkt 10 uM for 22h before harvesting. Subcellular fractionation was performed to obtain cytosol and membrane fractions. Proteins were solved by PAGE and immunoblotted. Anti-Akt, anti-pAkt, anti-AS160 and anti-pAS160 antibodies were used to assess phosphorylation state of both proteins. Bacterial Major Outer Membrane Protein was detected with Anti-MOMP antibodies. Anti-clathrin was used as membrane marker, while anti-actin, as cytosolic marker. **(E)** HeLa cells infected with *Ct* (MOI 1) were incubated with DMSO or iAkt (10 μM) from 2 h pi until fixation at 24 h pi. AS160 subcellular localization was detected with rabbit polyclonal anti-AS160 antibodies followed by Cy3-labeled IgG and then cells were analyzed by confocal microscopy. Asterisks point out inclusions. Bars represent 10 μm. **(F)** HeLa cells were depleted of AS160 and infected with *Ct* (MOI 1). Chlamydial inclusion area was analyzed by confocal microscopy. Data are the mean ± SEM of three independent experiments (^∗∗^*p* < 0.01) of 100 cells each. **(G)** AS160 knockdown was confirmed by western blot. Data are the mean ± SEM of three independent experiments (^∗∗∗^*p* < 0.01).

Akt acts on more than 35 substrates; being AS160 one of these downstream targets (Huang et al., 2018). AS160 is a GAP that promotes its cognate Rab GTPases to hydrolyze GTP and remain GDP loaded in their inactive state. AS160 can be phosphorylated on several serine/threonine residues; further Akt-dependent AS160 phosphorylation abrogates its GAP activity (Ishikura et al., 2006; Mafakheri et al., 2018). First, we determined if *C. trachomatis* infection was able to cause AS160 phosphorylation and/or modify AS160 expression along the entire bacterial developmental cycle. Phosphorylated AS160 (pAS160) was detected by an antibody that recognizes the phosphorylated residue at Ser-318, which is conveniently involved in the loss of GAP activity (Sano et al., 2003; Geraghty et al., 2007). By western blot, we assessed pAS160 and AS160 in HeLa cells infected with *C. trachomatis* for different periods of time. Consequently, we verified a readily observable phosphorylation of AS160 that, in the absence of fetal bovine serum, increased its magnitude along chlamydial infection (Figure 3A). To further confirm *C. trachomatis*-induced AS160 phosphorylation, we infected HeLa cells with alive or heat-inactivated bacteria; and at 2 h pi, we harvested the cells to detect pAS160 by western blot. Our results showed that viable *C. trachomatis* induces AS160 phosphorylation more efficiently than dead bacteria. There is a brief but consistent activation of Akt at the initial steps of phagocytosis, independently of the pathogen that is being internalized, or if it is dead or alive. Furthermore, this fact is also observed during the uptake of any type of beads. This Akt phosphorylation is related to the actin polymerization that it is needed for pseudopod extension at the phagocytic cup (Gu et al., 2003). As activated Akt phosphorylates AS160, dead bacteria-induced Akt activation produced AS160 phosphorylation at early stages of infection, as expected. However, pAS160 levels were substantially higher when cells were infected with alive bacteria (Figure 3B). To further connect *C. trachomatis*-induced Akt activation with AS160 phosphorylation, we explored whether Akt functions as a kinase for AS160. Uninfected and *C. trachomatis*-infected cells were treated with iAkt before analysis of pAS160 by western blot. pAS160 drastically decreased in the presence of iAkt, indicating that Akt is the main kinase responsible for AS160 phosphorylation during chlamydial infection (Figure 3C). Akt exerts its function on AS160 when they are both associated to the same membrane (Larance et al., 2005; Gonzalez and McGraw, 2009). In uninfected cells, AS160 is detached from membranes upon phosphorylation (Thong et al., 2007). For these reasons, we explored Akt/AS160 association to membranes in the context of *C. trachomatis* infection. Briefly, a subcellular fractionation to separate membranes from cytosol was performed in uninfected and infected cells. Then, proteins were separated by PAGE and immunobloted with specific antibodies. Clathrin was used as a membrane marker whereas actin indicated the cytosolic fraction. According to previous reports, actin was partially collected in the membrane fraction due to its association to the inclusion membrane (Kumar and Valdivia, 2008). Detection of the major outer membrane protein (MOMP) was indicative of bacteria. As expected by the findings developed above, we found less bacterial protein after iAkt treatment. Regarding Akt, infection triggered its phosphorylation, while the inhibitor blocked it. Interestingly, we found an increase in the amount of non-phosphorylated AS160 (AS160) associated to membranes, concominantly to Akt inhibition. Additionally, these results further confirmed that phosphorylated AS160 (pAS160) detached from membranes and thus we could not detect it on the membrane fraction (Figure 3D). To corroborate these findings, we analyzed by confocal microscopy AS160 subcellular localization in *C. trachomatis*-infected cells incubated in the absence or presence of the Akt inhibitor. In untreated cells, phosphorylation of AS160 caused its release from membranes, adopting a cytosolic distribution; whereas in the presence of iAkt, AS160 remained dephosphorylated and associated with internal membranes, as expected by its phosphorylation state (Kane et al., 2002; Larance et al., 2005; Peck et al., 2006). Confocal images showed smaller chlamydial inclusions, clearly decorated with AS160, in infected cells treated with iAkt (Figure 3E). Furthermore, to directly assess the participation of AS160 on chlamydial infection, HeLa cells, previously transfected with siRNA-AS160 or siRNA-Luciferase for 48 h, were infected with *C. trachomatis*. At 24 h pi, the percentage of infected cells and the growth of inclusions were determined by confocal microscopy (Figure 3F). Analysis of AS160 involvement in bacterial uptake showed that this protein does not participate in the internalization process (Supplementary Figure S3). AS160 silencing was confirmed by western blot (Figure 3G). Nonetheless the lack of effect of AS160 knockdown on the number of infected cells, our findings indicate that AS160 depletion causes a significant decrease in inclusion size. This finding is in agreement with previous reports pointing out that GTP/GDP cycling is essential for Rab function, implying the participation of AS160 in chlamydial inclusion growth.

### Rab14 Recruitment to Chlamydial Inclusions Is Interfered by Akt/AS160 Pathway Inhibition

**FIGURE 4:**
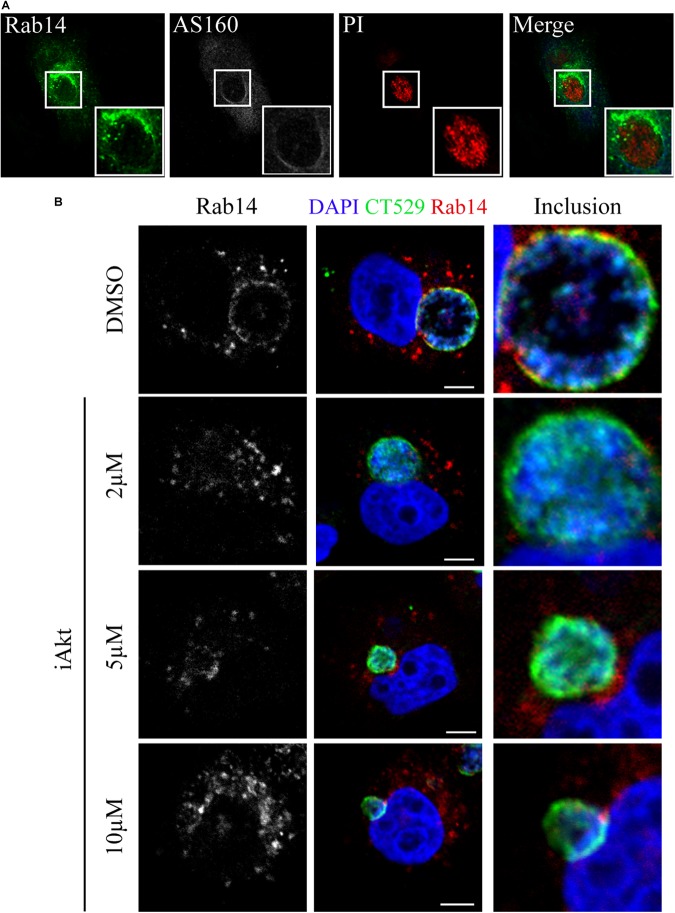
Rab14 recruitment to chlamydial inclusions is hindered by Akt/AS160 inhibition. **(A)** HeLa cells were infected with *Ct* (MOI 1) for 24 h. Immunodetection of endogenous AS160 with rabbit antibody followed by Cy5-coupled secondary antibodies. Detection of endogenous Rab14 was performed with mouse antibody followed by FITC-coupled secondary antibodies. Propidium iodide (PI) stained DNA. **(B)** Infected cells (MOI 1) were incubated with DMSO or increasing concentrations of iAkt (2, 5, and 10 μM) from 2 h pi until fixation at 24 h pi. Subcellular distribution of endogenous Rab14 was observed by confocal microscopy using mouse primary antibody followed by Cy3-conjugated anti-mouse secondary antibody. Inclusion membrane was detected using rabbit anti-CT529 antibody followed by FITC-conjugated anti-rabbit IgG. DAPI was used to stain DNA. Bars represent 10 μm. Images are representative of three independent experiments.

Akt-mediated phosphorylation of AS160 abrogates its GAP activity, leading to the prevailing active state of its downstream target Rab14 (Ishikura et al., 2006; Brewer et al., 2016). We have previously shown that *C. trachomatis* actively recruits Rab14 to the inclusion membrane along bacterial developmental cycle (Capmany and Damiani, 2010; Capmany et al., 2011). Furthermore, we have shown that GTP/GDP cycling is necessary to accomplish Rab14 function since GDP-bound mutant (GFP-Rab14 S25N) is retained at the Golgi apparatus in infected cells (Capmany and Damiani, 2010). Therefore, we decided to analyze by confocal microscopy the subcellular localization of both AS160 and Rab14 after *C. trachomatis* infection. Noteworthy, Rab14 was observed at the inclusion membrane region where AS160 was not present (Figure 4A). This finding is in line with the general concept that active non-phosphorylated AS160 is associated with membranes where it promotes the hydrolysis of GTP bound to Rab14, turning the Rab into its inactive soluble GDP-bound form, thus, favoring the release of Rab14 from the membrane. On the contrary, when AS160 is phosphorylated, it is detached from membranes and, consequently, it cannot exert its GAP activity; hence, Rab14 remains in its active GTP-bound state associated to the membrane for a longer time (Mafakheri et al., 2018). As expected, AS160 knock down detrimentally impacted on chlamydial inclusion development with the appearance of smaller vacuoles. Hence, impairment of GTP/GDP cycling affected Rab14 function in transport between compartments (Supplementary Figure S4). Although implied, our experiments did not show GTP loading of Rab14. Therefore, we confirmed Rab14 binding to GTP by its ability to recruit effectors like RUFY1 that only interacts with Rab14 GTP-bound form (Yamamoto et al., 2010). On the contrary, after iAkt treatment, thus, in the case of AS160 exerting its GAP activity, RUFY1 and Rab14 mostly display a cytosolic pattern (Supplementary Figure S5).

Next, we analyzed the effect of iAkt on Rab14 recruitment to chlamydial inclusions. Briefly, HeLa cells infected with *C. trachomatis* were treated 2 h later with increasing concentrations of iAkt or DMSO until fixation at 24 h pi. Endogenous Rab14 was immunodetected with a specific antibody and inclusion membrane was marked by labeling the bacterial protein CT529. Accordingly, Rab14 recruitment to the inclusion membrane was hindered by iAkt in a dose-dependent manner. Moreover, treatment with the highest concentration of Akt leads to the complete dissociation of Rab14 from the inclusion (Figure 4B). The loss of association with the inclusion membrane of Rab14 after iAkt treatment, strongly suggests that Akt/AS160 signaling pathway regulates vesicular transport controlled by this Rab in *C. trachomatis*-infected cells. Likely, *C. trachomatis* promotes the arrival to the inclusions of Rab14-vesicles by hijacking PI3K/Akt/AS160 signaling pathway.

### Chlamydial Inclusions Fail to Acquire Golgi-Derived Sphingolipids After Akt Inhibition

**FIGURE 5:**
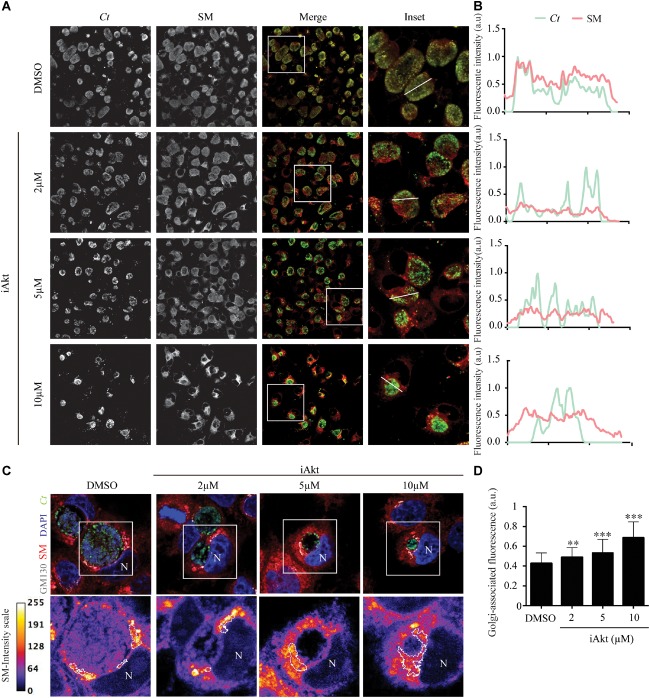
Sphingolipids transport to the inclusions is interfered by Akt inhibition. **(A)** Infected cells (MOI 2) were incubated with DMSO or increasing concentrations of iAkt (2, 5, and 10 μM) from 2 to 24 h pi. Before fixation, cells were incubated for 30 min with Ceramide-BODIPY at 4°C in serum-free DMEM. Then, cells were washed with PBS and incubated with DMEM supplemented with FBS for 30 min at 37°C. Finally, bacteria were detected with anti-MOMP coupled to FITC. **(B)** Intensity profiles obtained by scanning red (SM) and green (*Ct*) fluorescence along a line that crosses inclusions. **(C)** In experiments conducted as described in **(A)**, Golgi apparatus was detected with mouse anti-GM130 antibody followed by anti-mouse Cy5-coupled secondary antibody. Bacteria were detected with anti-MOMP coupled to FITC. DAPI stained nuclei (N) and bacterial DNA. Concentration and localization of sphingolipids are depicted in the bottom panels. Fire scale represents the fluorescence intensity associated to sphingolipids. Golgi apparatus is delimited with a white line. **(D)** Fluorescence intensity corresponding to sphingolipids at the Golgi apparatus was quantified using ImageJ. Data are representative of three independent experiments (^∗∗∗^*p* < 0.001, ^∗∗^*p* < 0.01).

Considering our previous findings that Rab14 mediates sphingolipids delivery to chlamydial inclusions (Capmany and Damiani, 2010) together with our current results showing that Akt inhibition decreases Rab14 recruitment to the inclusions, we predicted that transport of sphingolipids toward chlamydial inclusions would be affected by iAkt treatment. To prove this hypothesis, cells infected with *C. trachomatis* L2 (*Ct*) were incubated with increasing concentrations of iAkt from 2 h pi until the end of the experimental period. Fluorescent BODIPY TR ceramide, a sphingomyelin (SM) precursor, was used as a probe for sphingolipids trafficking (Hackstadt et al., 1995). Before fixation, cells were incubated with BODIPY TR ceramide for 30 min at 4°C, and then, the medium was renewed and cells were incubated for an additional 30 min period at 37°C. Finally, cells were fixed and bacteria were detected by direct immunofluorescence with a specific antibody against major outer membrane protein (MOMP) coupled to fluorescein (FITC). In untreated *C. trachomatis*-infected cells, there was a complete overlapping of the fluorescence corresponding to bacteria and SM. In addition to the decrease in bacterial growth and inclusion development, there was a significant reduction in sphingolipids acquisition by chlamydial inclusions after iAkt treatment in a dose-dependent manner (Figure 5A). At the right side of the images, representative profiles of fluorescence intensity are shown. Each profile was obtained by scanning green and red fluorescence intensities along a line that traverses the indicated inclusion (Figure 5B). In line with our hypothesis, Akt inhibition appears to cause sphingolipids retention at the periphery of chlamydial inclusions. To further analyze this finding, HeLa cells infected with GFP-expressing *C. trachomatis* (*Ct*-GFP) were treated as described in the experiment above, but in this case, we labeled the Golgi apparatus by detecting GM130, a structural Golgi-associated protein. Bacterial and eukaryotic DNA was stained with DAPI. Images show that SM did not reach the chlamydial inclusion, being retained at the Golgi apparatus after Akt inhibition (Figure 5C). Furthermore, blockage of SM departure from the Golgi apparatus was dependent on iAkt concentration. Golgi-associated SM fluorescence intensity was measured by confocal microscopy in every iAkt doses assayed (Figure 5D). Additionally, we used BODIPY FL ceramide that shifts fluorescence from 515 nm (green) to 620 nm (red) at increasing concentrations. Experiments were performed as described above for BODIPY TR ceramide. Golgi apparatus was detected with GM130 antibodies and DNA was stained with DAPI. With this different approach, we confirmed that Akt inhibition reduced the arrival of sphingolipids to the chlamydial inclusion and caused a readily observable enrichment of these lipids at the Golgi apparatus (Supplementary Figure S6).

**FIGURE 6:**
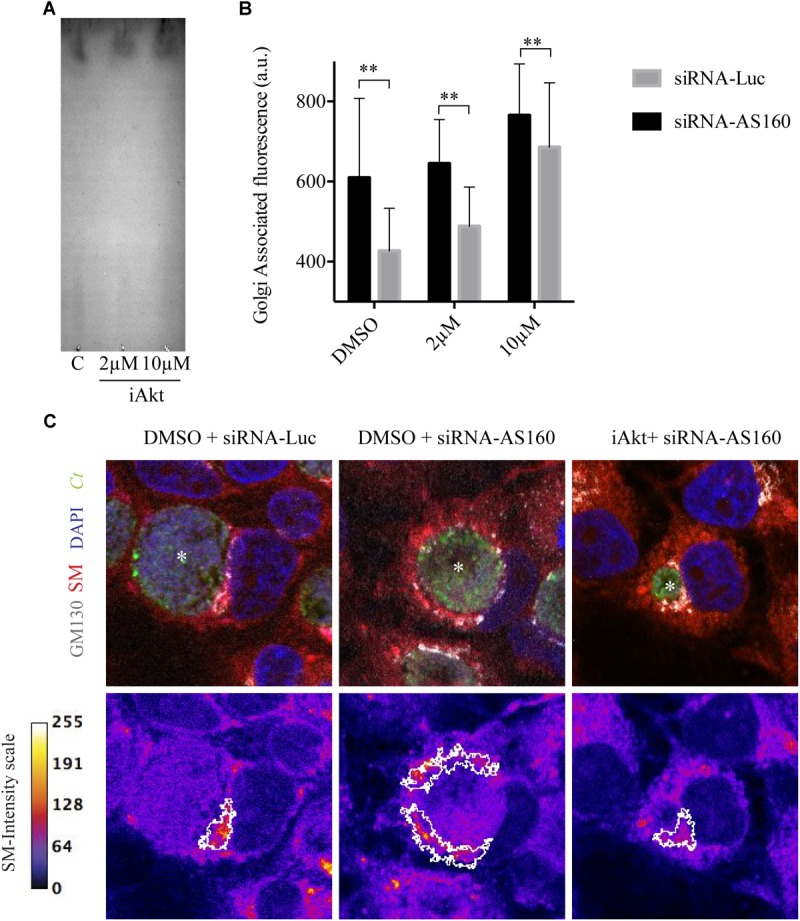
Disruption of the Akt/AS160 pathway causes retention of lipids at the Golgi apparatus. **(A)** Equal amounts of lipids extracted from infected cells incubated with DMSO, 2 μM or 10 μM iAkt were resolved by thin layer chromatography (TLC). A representative TLC from two independent experiments is shown. **(B)** HeLa cells were transfected with either siRNA-Luc or siRNA-AS160 and, 48 h later, infected with GFP-expressing *C. trachomatis* (MOI of 1). AS160-silenced and control cells were incubated with DMSO or increasing concentrations of iAkt (2 or 10 μM). After 24 h of infection and before fixation, cells were incubated for 30 min with BODIPY TR ceramide at 4°C in serum-free DMEM. Then, cells were washed with PBS and incubated with DMEM supplemented with FBS for 30 min at 37°C. Golgi apparatus was immunodetected in fixed cells with mouse anti-GM130 monoclonal antibody followed by Cy5 coupled anti-mouse secondary antibody. Bars show the average fluorescence intensity of lipids at the Golgi apparatus. ImageJ was used for this analysis. **(C)** Representative images of two independent experiments performed as indicated in **(B)**. In the upper panels, subcellular distribution of sphigolipids appears in red. GFP expressing bacteria are shown in green. Golgi was stained with mouse anti-GM130 followed by anti-mouse Cy5-coupled secondary antibody (white). In the lower panel, fire scale represents the fluorescence intensity associated to sphingolipids. White lines demarcate Golgi structures. Nuclei were stained with DAPI. Asterisks indicate inclusions.

Next, we analyzed whether Akt inhibitor modifies type or concentration of fluorescent lipids of cells incubated with BODIPY TR ceramide. By thin layer chromatography (TLC), we observed that there was the same fluorescent lipid species within untreated (DMSO) cells or in cells after treatment with 2 μM or 10 μM iAkt (Figure 6A). We further confirmed that the observed phenomenon was due to a trafficking defect by the analysis of lipids distribution and dynamics in AS160-depleted cells, in which Akt was not inhibited. Images showed that the fluorescent lipid was retained at the Golgi apparatus not only after treatment with the Akt inhibitor but also after AS160 silencing. Moreover, Akt inhibition in AS160-depleted cells did not significantly increase fluorescent lipid retention at the Golgi, indicating that Akt acts upstream from AS160 in this cascade (Figure 6B,C). Taken together, the lack of lipids within chlamydial inclusions is a consequence of its retention at the Golgi apparatus after Akt inhibition or AS160 silencing, constituting a trafficking defect and not an alteration of lipid metabolism.

**FIGURE 7:**
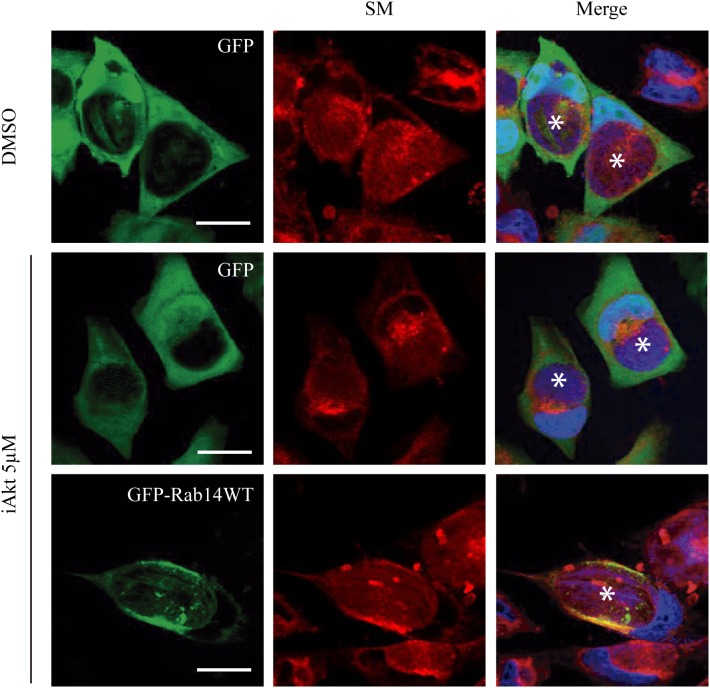
iAkt effect is overcome by Rab14 WT overexpression. HeLa cells were transfected with either GFP or GFP-Rab14 WT 24 h prior infection (MOI 5). At 2 h post infection, GFP expressing cells were treated with DMSO or iAkt 5 μM, and GFP-Rab14 WT expressing cells, with iAkt 5 μM until fixation at 24 h pi. Nuclei were stained with DAPI. Asterisks point out inclusions. Bars represent 10 μm. Representative of two independent experiments.

Finally, involvement of Akt activity in the control of Rab14-mediated delivery of sphingomyelin to the chlamydial inclusions, was evaluated in a rescue assay. Briefly, HeLa cells were transfected with either GFP or GFP-Rab14WT 24 h prior infection. Cells were infected for 24 h (MOI 5). At 2 h pi, GPF-expressing cells were incubated with the vehicle (DMSO) or 5 μM iAkt for 22 h; and GFP-Rab14 WT- expressing cells, with 5 μM iAkt (22 h). As expected, Akt inhibition caused a decrease in the arrival of sphingolipids to the inclusions in GFP-overexpressing cells. In contrast, Rab14 overexpression overcame iAkt effect. Thus, we observed higher fluorescence intensity within inclusions in Rab14-overexpressing cells, indicating that a higher amount of lipids reached the inclusions (Figure 7).

Taken together, our findings further support the notion that *C. trachomatis*, by hijacking Akt/AS160 signaling pathway, promotes the redirecting to the inclusion of Golgi-derived Rab14-vesicles that transport endogenously synthesized sphingolipids. Therefore, Akt inhibition or AS160 depletion hinders Rab14-controlled SM delivery from the Golgi to chlamydial inclusions.

### Akt/AS160/Rab14 Axis Blockage Impairs Chlamydial Replication and Infectivity

**FIGURE 8:**
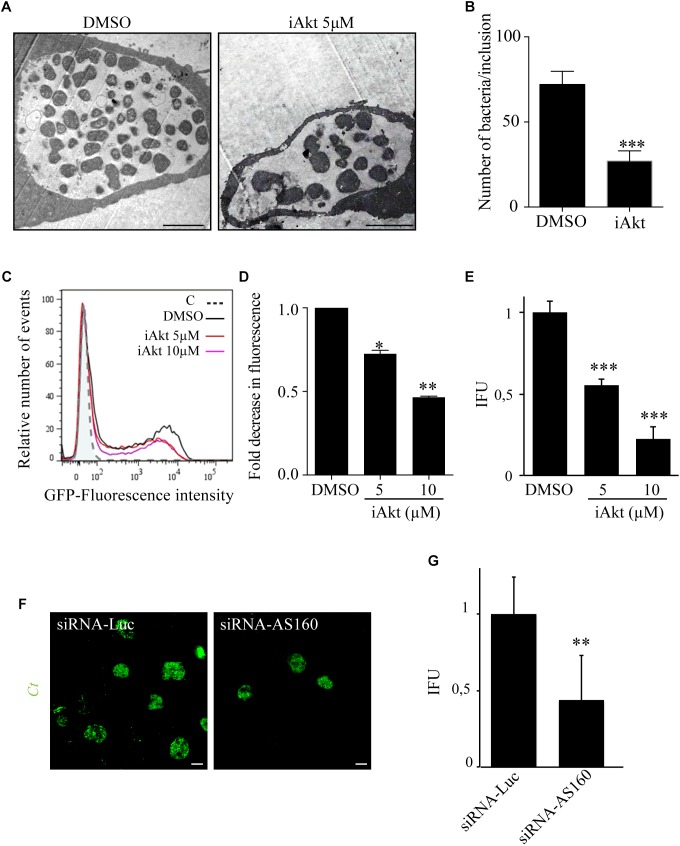
Akt inhibition impairs chlamydial replication and infectivity **(A)** HeLa cells infected with *Ct* (MOI 1) were treated with DMSO or iAkt (5 μM) from 2 h pi until fixation at 24 h pi. Cells were processed for transmission electron microscopy (TEM). **(B)** Quantification of the number of bacteria per inclusion by TEM. At least 30 cells of each experimental condition were analyzed (^∗∗∗^*p* < 0.001). **(C)** HeLa cells were infected with *Ct*-GFP (MOI 0.5) and treated with DMSO or iAkt (5 or 10 μM) from 2 h pi until the end of the experimental period. Cells were fixed at 24 h pi and analyzed by flow cytometry. **(D)** Quantification of green fluorescence (*Ct*) associated with infected cells. Results are expressed in relation to untreated infected cells. Data are representative of three independent experiments (^∗^*p* < 0.05, ^∗∗^*p* < 0.01). **(E)** HeLa cells infected with *Ct* (MOI 1) were incubated either with DMSO or increasing doses of iAkt (2, 5, or 10 μM). At 48 h pi, cells were lysed and the harvested infectious progeny was titrated in serial dilutions on HeLa cells. Bacteria were immunodetected with an anti-MOMP antibody coupled to FITC and inclusion forming units (IFU) were determined. Bars and the error bars represent mean ± SEM (^∗∗^*p* < 0.01, ^∗∗∗^*p* < 0.001). Data are representative of three independent experiments. **(F)** HeLa cells were transfected with either siRNA-luciferase or siRNA-AS160. After 24 h, cells were infected with *Ct* (MOI 1) for 48 h before lysis. Harvested EBs were titrated in serial dilutions on HeLa cells. Bacteria were detected with an anti-MOMP antibody coupled to FITC. Confocal images are representative of three independent experiments. **(G)** Infectious progeny harvested from cells was assessed by IFU assays. Results are expressed as IFUs per microliter. Data are representative of three independent experiments (^∗∗^*p* < 0.01).

Host sphingolipids are crucial for *C. trachomatis* development and their transport to chlamydial inclusions is regulated by the Akt/AS160/Rab14 cascade, therefore, we analyzed the impact of Akt inhibition on overall bacteria fitness. First, HeLa cells infected with *C. trachomatis* L2 (*Ct*) for 24 h and treated with 5 μM iAkt for the last 22 h were analyzed at the ultrastructural level by electron microscopy. We found a remarkable decrease in both, the size of inclusions and the number of bacteria within inclusions, in infected cells treated with iAkt compared to untreated cells (Figure 8A,B). Interestingly, we noticed the presence of aberrant bacterial forms in iAkt-treated cells (Figure 8A), similar to those found under sphingolipids deprivation or after overexpression of inactive Rab14-GDP mutant protein (Robertson et al., 2009; Capmany and Damiani, 2010; Capmany et al., 2011). Current findings are consistent with our previous results, in as much as this pathway is inhibited, the recruitment of Rab14 to the inclusion decreases and consequently sphingolipids supply to the inclusion is deficient, generating the appearance of aberrant bacteria. Next, we confirmed the detrimental impact on bacterial replication of Akt inhibition by flow cytometry. Briefly, HeLa cells were infected with GFP-*Ct* for 24 h and increasing concentrations of iAkt were added 2 h pi until the end of the experimental period. A decrease in green fluorescence associated with infected cells correlates with a reduced amount of GFP-*Ct* within the cells which is indicative of less bacterial replication (Figure 8C,D). Concomitantly, we analyzed the effect of Akt inhibition on the generation of chlamydial progeny. Thus, we performed Inclusion Forming Units (IFUs) assays in the absence or presence of increasing concentrations of iAkt to evaluate the contribution of this kinase on the yield of infectious particles. Briefly, HeLa cells were infected with *C. trachomatis* and 2 h later, cells were incubated with increasing iAkt doses or DMSO and lysed at 48 h pi to harvest infectious EBs. Then, cell lysates were used to infect monolayers of HeLa cells in serial dilutions. After 24 h, these new round of infected cells were fixed and the number of inclusions was quantified by confocal microscopy. Inclusions were detected with an anti-MOMP antibody coupled to FITC and chlamydial infectious progeny was calculated. As expected, inhibition of Akt significantly reduced bacterial infectivity (Figure 8E). To further confirm the involvement of AS160 on chlamydial replication and infectivity, HeLa cells, previously transfected with siRNA-AS160 or siRNA-Luciferase, were then infected with GFP-*Ct* for 48 h before cell lysis. Then, EBs harvested from cell lysates were titrated in serial dilutions on HeLa cells (Figure 8F,G). Although AS160 silencing did not affect bacterial internalization, it had a detrimental effect on bacterial replication, as observed in images taken by confocal microscopy (Figure 8F). As expected, AS160 depletion significantly decreased the yield of infectious particles assessed by IFU assays (Figure 8G).

Taken together, our findings indicate that *C. trachomatis*-activated Akt/AS160/Rab14 pathway is critical to ensure sphingolipids supply to chlamydial inclusions and consequently to warrant chlamydial replication and development. Therefore, inhibition of Akt or downstream members of the Akt/AS160/Rab14 axis may limit chlamydial infection.

## Discussion

**FIGURE 9:**
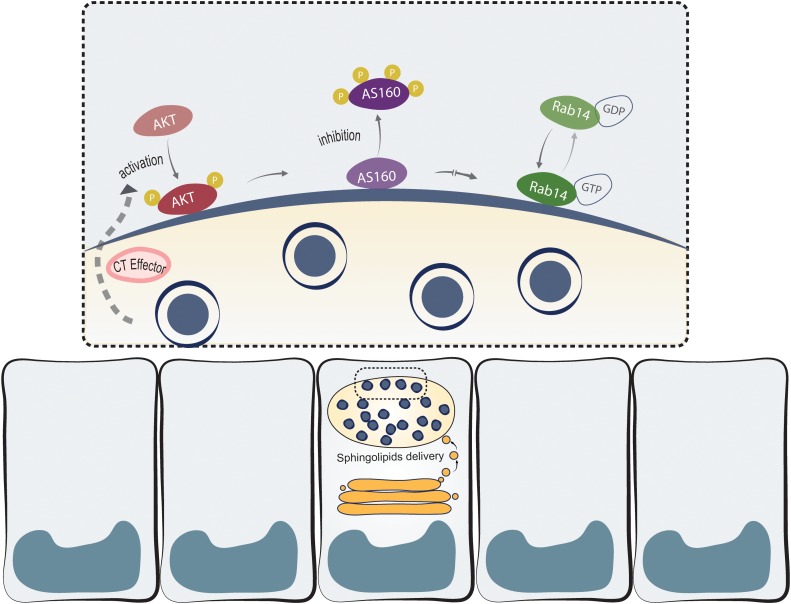
*Chlamydia trachomatis* hijacks Akt/AS160/Rab14 cascade to acquire Golgi-derived sphingolipids. *Ct* phosphorylates and recruits pAkt to the inclusion membrane by one or more cognate effectors (CT effector). Upon Akt activation, AS160 is inactivated by phosphorylation and it is detached from the inclusion membrane. Hindered AS160 GAP-activity enables Rab14 to remain in its GTP-bound membrane-associated active form. This, in turn, favors the arrival to chlamydial inclusions of sphingolipids synthesized at the Golgi apparatus through a Rab14-controlled vesicular transport.

*Chlamydia trachomatis*, like other obligate intracellular bacteria, needs the host machinery to replicate and survive (Bastidas et al., 2013; Damiani et al., 2014). Lipids acquisition from host cells is fundamental for both bacterial replication and inclusion membrane growth. Actually, *C. trachomatis* is able to recruit host lipid synthesis machinery at the inclusion membrane (Derré et al., 2011; Elwell et al., 2011) and to piracy lipids from TGN-derived vesicles (Hackstadt et al., 1996; Carabeo et al., 2003), multivesicular bodies (Beatty, 2006, 2008; Gambarte Tudela et al., 2015) and lipid droplets (Kumar et al., 2006; Cocchiaro et al., 2008). An extensively exploited strategy used by this bacterium is the recruitment of specific Rab proteins to subvert host vesicular trafficking (Damiani et al., 2014); nonetheless, the molecular mechanisms underlying the recruitment of Rab proteins are largely unknown. We have reported that *C. trachomatis* intercepts Rab14-vesicles, derived from the Golgi apparatus and in transit to the plasma membrane, to acquire endogenously synthesized sphingolipids (Capmany and Damiani, 2010; Capmany et al., 2011). Here, we showed the participation of Akt/AS160 signaling pathway in sphingolipids delivery to chlamydial inclusions. Our working model is displayed in Figure 9. This is the first report to link a phosphorylation cascade, initiated by this bacterium, and the recruitment of a Rab protein to the inclusion membrane with the subsequent redirection of Golgi-derived sphingolipids to chlamydial inclusion.

Recent evidence pointed out that TepP (translocated early phospho-protein), a chlamydial effector located at the inclusion membrane, recruits PI3K and the signaling adaptor protein CrkL forming a complex. TepP is a protein involved in bacterial replication and activation of type I IFN genes. Although translocation of TepP is not related to Akt activation, it could be involved in its recruitment (Carpenter et al., 2017). PI3K acts upstream from Akt in this signaling pathway, therefore, this report reinforces our findings. In this study, we found Akt is phosphorylated throughout the entire developmental cycle, standing out three phosphorylation peaks: at the beginning (2 h pi), at the middle (8 h pi), and at late stages (36 h pi) of the cycle. At the peaks, pAkt doubled or tripled the level of Akt phosphorylation in uninfected cells. However, total Akt expression remained unaltered in *Chlamydia*-infected cells. Furthermore, we showed a strong redistribution of endogenous pAkt after infection, which concentrated at the perinucleus where the bacteria were located. In agreement, several reports account for Akt phosphorylation after chlamydial infection, however, differences in phosphorylation dynamics could be due to different *Chlamydia* species or serovars, experimental conditions or host cell type (Verbeke et al., 2006; Rajalingam et al., 2008; Gurumurthy et al., 2010; Patel et al., 2014; Subbarayal et al., 2015). PI3K/Akt signaling pathway participates in multiple cellular processes such as apoptosis resistance and cell survival, glucose metabolism, cell growth and proliferation, actin recruitment and phagosome formation, among others (Innocenti et al., 2003; Engelman et al., 2006; Yeo et al., 2015; Zhao et al., 2015). We focused our efforts to elucidate the link between PI3K/Akt cascade and intracellular transport of sphingolipids in *C. trachomatis*-infected cells. One of the substrates of Akt is AS160 (Akt Substrate of 160 kDa), a GTPase Activating Protein (GAP) for several Rabs. In general, Rab GTP/GDP cycling is regulated by a delicate equilibrium between GTPase activating proteins (GAPs) and guanine nucleotide exchange factors (GEFs), controlling Rab subcellular localization and displacement between cellular compartments. Proper translocation from a donor to an acceptor membranous compartment requires a fine tune controlled GTP/GDP cycling (Voss et al., 2019). Akt phosphorylates and, consequently, inactivates AS160 when they are both associated to the same membrane (Larance et al., 2005; Gonzalez and McGraw, 2009). Phosphorylated AS160 detaches from membranes and loses its GAP activity; hence, the target Rab remains in its active, membrane-associated, GTP-bound state for longer (Mîinea et al., 2005; Thong et al., 2007). By membrane/cytosol fractionation, we confirmed that Akt activation in the context of *C. trachomatis* infection triggers AS160 phosphorylation and its release from membranes. Consequently, Akt inhibition increases AS160 association with membranes where it enhances the intrinsic Rab GTPase activity of the cognate Rab.

AS160 is GAP for Rab2, Rab8A, Rab10, and Rab14 (Kane et al., 2002; Sano et al., 2003; Mîinea et al., 2005). Rab10 associates with chlamydial inclusions in a species-dependent manner, only being found on *Chlamydia pneumoniae* inclusions (Cortes et al., 2007). At present, Rab2 and Rab8 involvement in chlamydial infections have not been investigated. Since AS160 modulates Rab14 GTP/GDP cycling and *C. trachomatis* hijacks Rab14-controlled intracellular trafficking; an interesting challenge was to determine if there was a relationship between Akt activation and vesicular transport in *C. trachomatis*-infected cells. We observed that AS160, like Akt, is phosphorylated during the entire developmental cycle, but unlike Akt, AS160 displays a steady level of phosphorylation over time. This may indicate a phosphorylation/dephosphorylation turnover that favors AS160 phosphorylated form, or it could be likely attributed to inactivation of a putative AS160 phosphatase by *C. trachomatis*. Alternatively, other kinases, besides Akt, may be involved in AS160 phosphorylation. Actually, it has been described that PKC and AMPK phosphorylate AS160 in muscle cells to induce GLUT4 translocation (Thong et al., 2007). Nevertheless, our findings indicate that *C. trachomatis*-induced Akt activation is the main via of AS160 phosphorylation; since pAS160 drastically decreased after Akt inhibition. We confirmed these results by confocal microscopy. In infected cells, AS160 displayed a cytosolic distribution, as expected by its phosphorylation provoked by activated Akt; accordingly, AS160 was recruited to chlamydial inclusions under iAkt treatment. Moreover, we found Rab14 associated with the inclusion membrane in those domains where AS160 was not present. In agreement, treatment of infected cells with an allosteric Akt inhibitor (iAkt) hindered Rab14 recruitment to chlamydial inclusions in a dose-dependent manner.

Akt/AS160/Rab14 axis has been extensively studied in adipocytes and muscle cells, in which, insulin stimulus results in GLUT4 translocation to the plasma membrane. Signaling cascade begins with Akt phosphorylation which in turn phosphorylates and inactivates AS160, favoring Rab14-controlled GLUT4 translocation (Kane et al., 2002; Sano et al., 2003; Mîinea et al., 2005; Ishikura et al., 2006; Klip et al., 2014). Similarly, through the activation of the same signaling pathway, Similiki Forest virus, Ross River virus and *Mycobacterium tuberculosis* alter glucose and lipid metabolism of the host cell by the translocation of some GLUT isoforms from an intracellular pool to the plasma membrane (Dasgupta and Rai, 2018; Mazzon et al., 2018). In line with these findings, we have previously shown that GTP/GDP cycling is necessary to accomplish Rab14 function since the GDP-bound mutant (GFP-Rab14 S25N) is retained at the Golgi apparatus in infected cells (Capmany and Damiani, 2010). Furthermore, Rab14 silencing reduces chlamydial infectious progeny in a similar extent to overexpression of the inactive GDP-bound mutant form of Rab14 (Capmany and Damiani, 2010).

Our studies showed that Akt inhibition impairs *C. trachomatis* growth and replication denoted by a decrease in chlamydial inclusion size, bacterial multiplication, and infectivity assessed by different experimental approaches like confocal microscopy, electron microscopy, flow cytometry, and IFU assays. Similar results were obtained in AS160-depleted cells, confirming that Rab14 GTP/GDP cycling is necessary for optimal bacterial fitness. Supporting these findings, it has been reported that *Salmonella typhimurium* and *Staphylococcus aureus* avoid degradation by manipulating Akt/AS160/Rab14 axis to prevent or delay phagosome-lysosome fusion (Kuijl et al., 2007; Casanova, 2017; Lacoma et al., 2017).

Summarizing, we have determined that the activation of Akt induced by chlamydial infection leads to phosphorylation and inactivation of AS160 and the subsequent recruitment to inclusions of Rab14. The remaining question to address was about the benefit that *C. trachomatis* obtains by activating the Akt/AS160/Rab14 pathway. Giving consideration to our previous results, which demonstrates that *C. trachomatis* usurps Rab14-controlled vesicular transport to piracy Golgi-derived sphingolipids (Capmany and Damiani, 2010), and these new results that show an impaired recruitment of Rab14 after iAkt treatment in a dose-dependent manner, we hypothesized that *C. trachomatis* could activate Akt/AS160 pathway to ensure sphingolipids delivery to chlamydial inclusions. By the use of BODIPY-Ceramide as a sphingolipid precursor, we observed that Akt inhibition caused the retention of these lipids at the periphery of inclusions, particularly at the Golgi apparatus. Consistently, a reduced amount of sphingolipids were found inside inclusions after iAkt treatment in a dose-dependent manner. By electron microscopy, we observed not only smaller inclusions with less *Chlamydia* but also the appearance of aberrant bacterial forms after Akt inhibition. Similarly, aberrant bacteria were also generated by lipid deprivation, overexpression of inactive GDP-bound Rab14 mutant or Rab14 silencing (van Ooij et al., 2000; Capmany and Damiani, 2010). Involvement of Akt activity in the control of Rab14-mediated delivery of sphingomyelin to the chlamydial inclusions was confirmed in a rescue assay, in which overexpression of Rab14 overcame iAkt effects. Altogether, the activation of Akt not only facilitates *C. trachomatis* internalization and inhibition of the host cell apoptosis but also promotes the redirecting of post-Golgi vesicles full of sphingolipids to chlamydial inclusions. In addition, AS160 depletion generated chlamydial inclusions of smaller size and caused sphingolipids retention at the Golgi apparatus, confirming that impairment of GTP/GDP cycling reduces Rab14-controlled sphingolipids delivery to chlamydial inclusions. Further, we demonstrated that ceramide to sphingomyelin conversion was unaffected by Akt inhibitor or AS160 silencing, therefore, the decrease in the amount of sphingolipids within inclusions is a consequence of its retention at the Golgi apparatus, constituting a transport defect and not a metabolic alteration.

In line with our findings, it has been shown that *S. typhimurium* effector SopB promotes intracellular survival by blocking phagosome-lysosome fusion through the Akt/AS160/Rab14 pathway (Kuijl et al., 2007). Furthermore, multidrug-resistant *M. tuberculosis* usurps the same signaling pathway to impede phagosome maturation (Lacoma et al., 2017). The Akt inhibitor used in the present research impaired infections caused by these intracellular pathogens and opened a new road for the treatment of these difficult to eradicate bacteria (Kuijl et al., 2007). Many viruses have evolved mechanisms to manipulate host cell signaling pathways to generate a permissive intracellular environment for replication. Among them, herpesviruses (VHS) which express multiple proteins that hijack PI3K/Akt signaling pathway to optimize virus entry, replication, latency, reactivation, and modulation of host immune responses (Cheshenko et al., 2013; Liu and Cohen, 2015). Interestingly, miltefosine, which blocks Akt phosphorylation, inhibits VHS plaque formation (Cheshenko et al., 2013). In addition, human immunodeficiency virus (HIV) Tat protein promotes the stress-induced activation of the PI3K/Akt signaling cascade to favor survival of infected macrophages. Hence, PI3K/Akt inhibitors could be effective to eliminate HIV-infected macrophages by apoptosis (Chugh et al., 2008).

Current antibiotics directly target microorganisms rather than pathogen–host cell relationship. However, the emergence of multidrug-resistant bacteria pushes up the development of new pharmacological tools that complement actual therapies. A major challenge in the generation of new antimicrobial agents is the poor knowledge of the complex interaction between bacteria and host cells that leads to intracellular survival of the microorganism. In this regard, substantial advances have been achieved in the use of inhibitors of the PI3K/Akt pathway that were initially designed as anti-tumoral drugs to limit infections. Our findings indicate that an allosteric isoform-specific Akt inhibitor, iAkt, is effective to restrict *C. trachomatis* growth and replication, to limit chlamydial inclusion development and to reduce bacterial progeny and overall infectivity in a dose-dependent manner. Furthermore, downsizing of inclusions and impaired bacterial replication by iAkt are not attributable to direct drug toxicity to the cells or the bacteria since iAkt effects are reversible; therefore, Akt inhibitor acts as a bacteriostatic agent rather than a bactericidal one at the doses assayed. Further research is necessary to fully characterize chlamydial proteins involved in the manipulation of Akt/AS160/Rab14 cascade in order to design specific targeted molecular therapies to control infections caused by this widespread intracellular pathogen.

## Data Availability

The datasets generated for this study are available on request to the corresponding author.

## Author Contributions

AC, JGT, MAB, and MTD substantially contributed to the conception and design of the work. AC, JGT, and MAB acquired data. AC, JGT, MAB, and MTD analyzed the results and interpreted the data. AC, JGT, MAB, and MTD drafted and/or critically revised the manuscript. MTD contributed reagents and funding to support research. All authors approved the version to be published and agreed to be accountable for all aspects of the work.

## Conflict of Interest Statement

The authors declare that the research was conducted in the absence of any commercial or financial relationships that could be construed as a potential conflict of interest.
