# Supplementary material for: Akt/AS160 Signaling Pathway Inhibition Impairs Infection by Decreasing Rab14-Controlled Sphingolipids Delivery to Chlamydial Inclusions
Source: Front Microbiol. 2019 Apr 3;10:666. doi: 10.3389/fmicb.2019.00666 (PMC6456686; doi:10.3389/fmicb.2019.00666)
Supplement: Supplementary file 1 [file Data_Sheet_1.PDF]

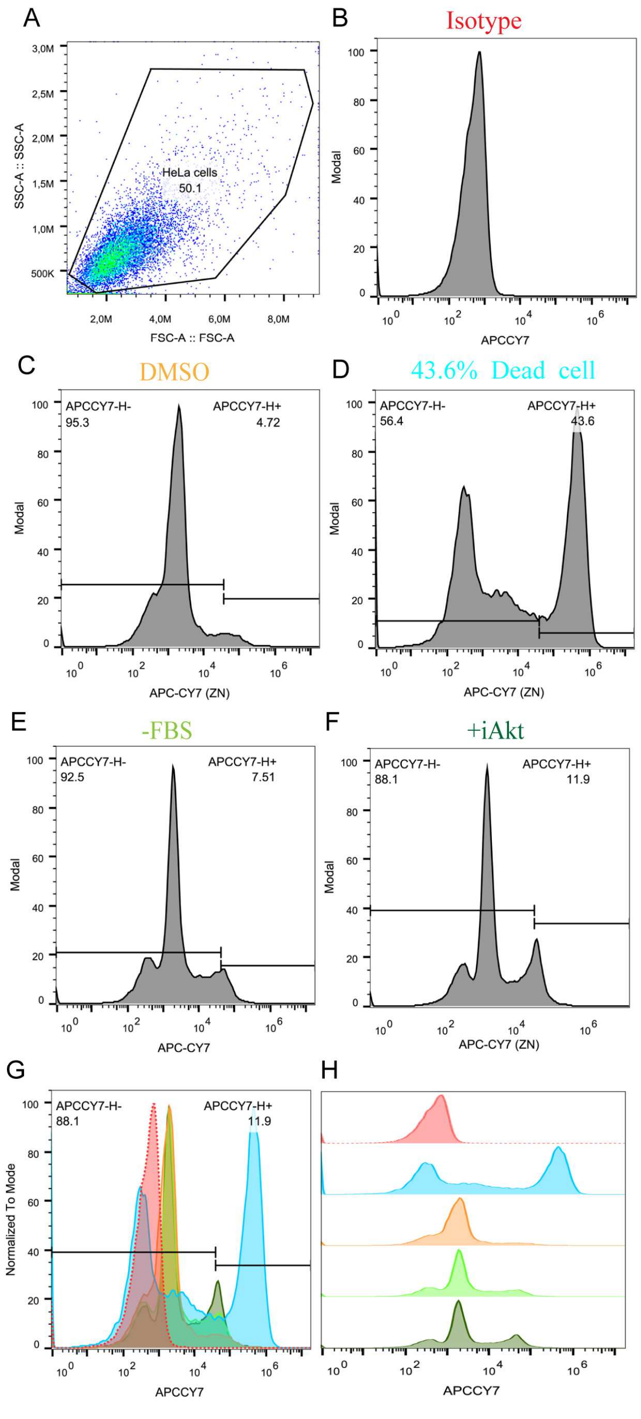

Supplementary Figure S1: Cell viability is unaffected by FBS starvation or iAkt treatment. **(A)** Selected HeLa cells population. **(B)** Autofluorescence control. **(C-F)** Cells incubated with Zombie NIR fixable kit were analyzed by flow cytometry. **(C)** Infected HeLa cells treated with DMSO for 24 h. **(D)** HeLa cells infected with a mix of live and dead (fixed) bacteria. **(E and F)** Infected HeLa cells FBS-starved for 24 h or treated with 10μM iAkt for 24 h. **(G and F)** Overlay plots of histograms.

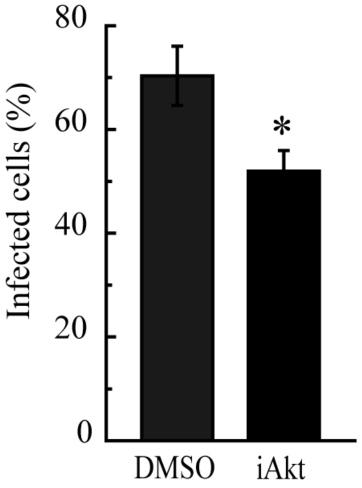

Supplementary Figure S2. Akt inhibition reduces chlamydial entry into host cells. HeLa cells were incubated with iAkt (5 μM) or DMSO, 2 h before infection (MOI 1). Percentage of infection was evaluated by confocal microscopy at 24 h pi. Data are the mean ± SEM of three experiments (*$p < 0.05$).

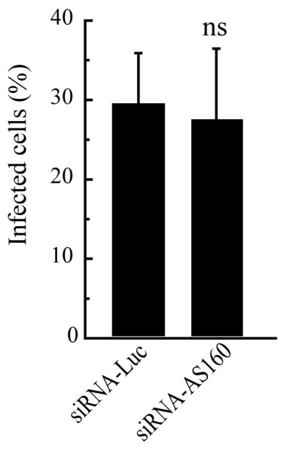

Supplementary Figure S3. AS160 is not involved in chlamydial uptake. AS160-depleted and control HeLa cells were infected (MOI 0.5) and 24 h, the percentage of infection was assessed by confocal microscopy. Data are the mean ± SEM of three experiments (ns: not significant).

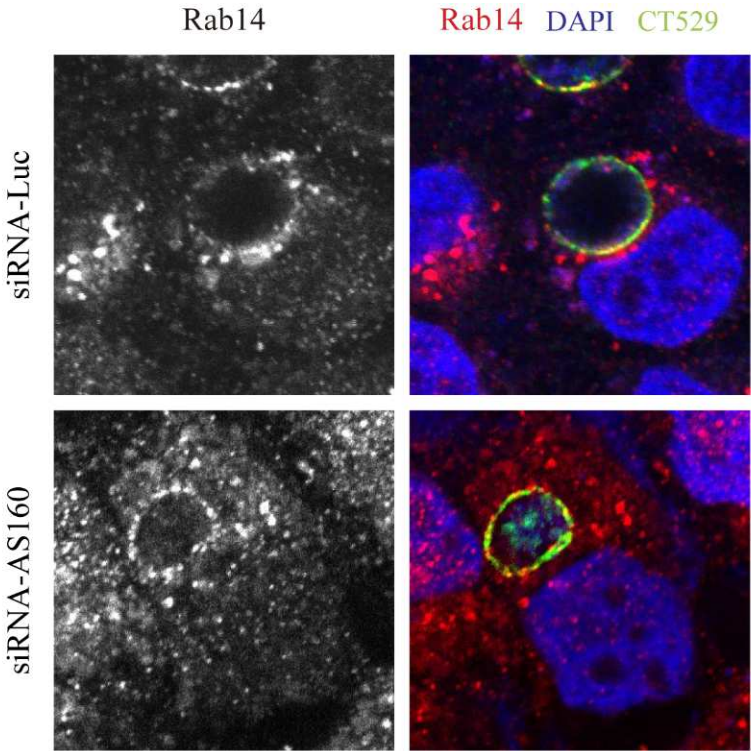

Supplementary Figure S4: Upon AS160 knockdown, Rab14 is retained at the inclusion membrane. HeLa cells were transfected with either siRNA-Luc or siRNA-AS160. At 48 h after transfection, cells were infected with *C. trachomatis* (MOI 1) for 24 h. After fixation, endogenous Rab14 was immunostained with monoclonal mouse anti-Rab14 followed by Cy3-coupled anti mouse secondary antibody (red). Inclusion membrane was detected with rabbit anti-CT529 antibody followed with FITC-coupled anti-rabbit antibody. DNA was stained with DAPI. Representative of two independent experiments.

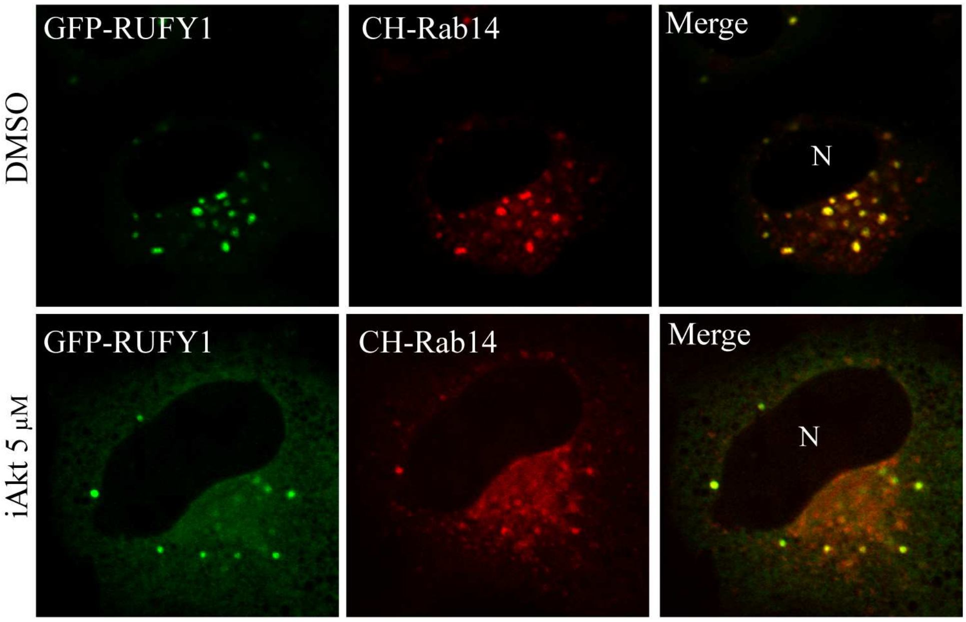

Supplementary Figure S5. GTP-bound Rab14 colocalizes with RUFY1. HeLa cells were cotransfected with GFP-RUFY1 and CH-Rab14. Cells were incubated for 4 h with DMSO or iAkt (5 μM) before confocal imaging. N indicates nuclei.

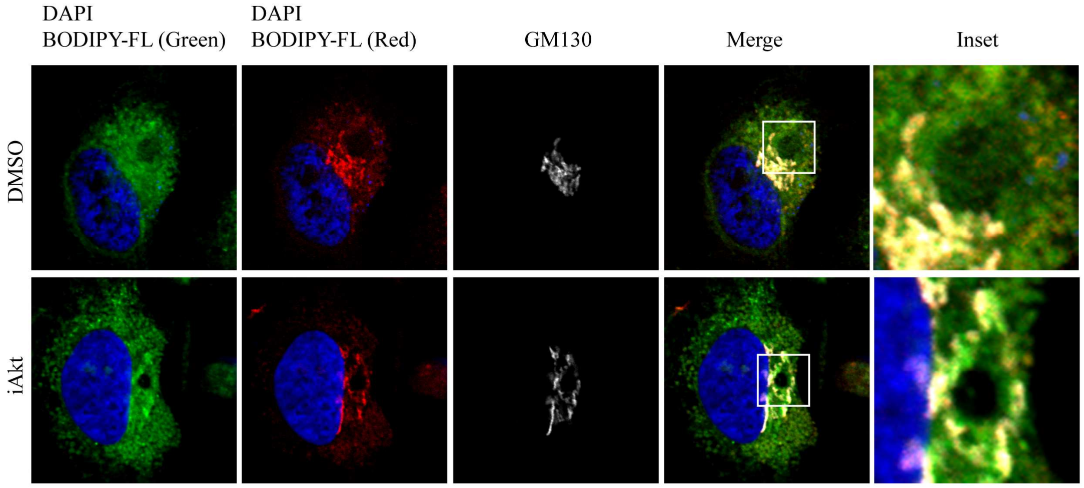

Supplementary Figure S6: BODIPY FL ceramide concentrates at the Golgi under iAkt treatment. After 2 h of infection, HeLa cells were treated with DMSO or 10 µM iAkt. At 10 h pi, cells were incubated for 30 min with BODIPY FL ceramide at 4° C in serum-free DMEM. Then, cells were washed with PBS and incubated with DMEM supplemented with FBS for 30 min at 37° C. BODIPY FL ceramide shifts from green to red fluorescence at increasing concentrations. After fixation, Golgi aparatus was immunodetected with mouse anti-GM130 monoclonal antibody followed by Cy5-coupled anti-mouse antibody.
